# Low molecular weight heparin calcium sequential rivaroxaban for the treatment of cerebral venous sinus thrombosis and cortical venous infarction in children with nephrotic syndrome: case report

**DOI:** 10.3389/fped.2025.1566114

**Published:** 2025-05-26

**Authors:** Xiumin Zhang, Juan Yang, Cuicui Guo, Jingcai Wang

**Affiliations:** Department of Pediatric, Liaocheng People’s Hospital, Liaocheng, Shandong, China

**Keywords:** rivaroxaban, cerebral venous sinus thrombosis, infarction, children, nephrotic syndrome

## Abstract

Cerebral venous sinus thrombosis (CVST), though exceedingly rare, represents a serious complication in pediatric patients with nephrotic syndrome (NS). We describe an 11-year-old male with steroid-resistant nephrotic syndrome (SRNS) maintained on prednisone/tacrolimus therapy who subsequently developed CVST. The patient manifested respiratory/gastrointestinal symptoms (cough, diarrhea) followed by neurologic deterioration featuring headache, seizures, and altered consciousness. Magnetic resonance imaging (MRI) and magnetic resonance venography (MRV) confirmed transverse sinus thrombosis with cortical venous infarction. A 5-day course of low-molecular-weight heparin (LMWH) followed by rivaroxaban achieved safe thrombus resolution, supporting this anticoagulation protocol in pediatric NS.

## 1 Introduction

NS, the most prevalent childhood glomerular disorder, confers a prothrombotic state with thromboembolism representing a critical complication. Current evidence-based estimates from large-scale meta-analyses indicate a 3.6%–4.9% incidence of thromboembolic events in pediatric primary NS, with venous thromboembolism (VTE) predominating over arterial events (1, 2). The common types of VTE include deep vein thrombosis of the lower limbs, renal venous thrombosis and pulmonary embolism (3). The precise prevalence of CVST in pediatric NS remains undefined, with a single Chinese cohort study (*n* = 1,995) reporting an incidence of 0.3% (4). Despite its rarity, CVST represents a critical neurosurgical emergency due to its potential progression to venous infarction or intracranial hemorrhage without prompt anticoagulation. The current anticoagulation regimen involves LMWH as initial therapy, with transition to vitamin K antagonists (e.g., warfarin) (5).

Direct oral anticoagulants (DOACs) can exert anticoagulant effects through direct inhibition of coagulation factor Xa or thrombin (coagulation factor IIa). The oral administration route, availability of formulations suitable for children, broad therapeutic window, prompt onset and offset of action, steady pharmacokinetics and pharmacodynamics, lower incidence of drug and food interactions and minimal or no need for monitoring bring a great advantage to DOACs (6–8). Rivaroxaban, a direct factor Xa inhibitor, received regulatory approval in 2021 from the European Medicines Agency and Health Canada for VTE management in pediatric populations (0–17 years), including catheter-related thrombosis and cerebral venous thrombosis (CVT). In 2022, rivaroxaban was approved in China for the treatment and secondary prevention of VTE in pediatric patients (≥30 kg) after at least 5 days of parenteral anticoagulation. Current evidence on rivaroxaban use in pediatric NS remains limited, with only three published studies identified, and no data available on its efficacy or safety in NS complicated by CVST (9–11). We report a pediatric case of NS complicated by CVST that was successfully managed with a short-course LMWH followed by long-term rivaroxaban therapy.

## 2 Case report

A previously diagnosed 11-year-old male with minimal change disease presenting as SRNS was admitted with disease exacerbation. Initially treated with prednisolone (20 mg TID) and tacrolimus (1 mg q12h) added at day 38, he developed recurrent edema 8 days prior to admission. Progressive symptoms emerged, including cough (5 days pre-admission), watery diarrhea (2 days pre-admission), and frontal headache (1 day pre-admission). Ten hours before admission, he experienced generalized tonic-clonic seizures followed by persistent unconsciousness. Initial management at a local hospital included intravenous mannitol (120 ml), penicillin (4 million units), and electrolyte replacement, yet seizure activity recurred three times despite intervention.

On physical examination, his Glasgow coma scale was 11(3 + 4 + 4). He had severe generalized edema, accompanied by asymmetrical cephalic edema that was more pronounced on the left side. There was a reduction in the breath sound detected in the right lower lung region. Active borborygmi were detected on abdominal auscultation. Meningeal irritation signs and pathological signs were both negative.

**Figure 1:**
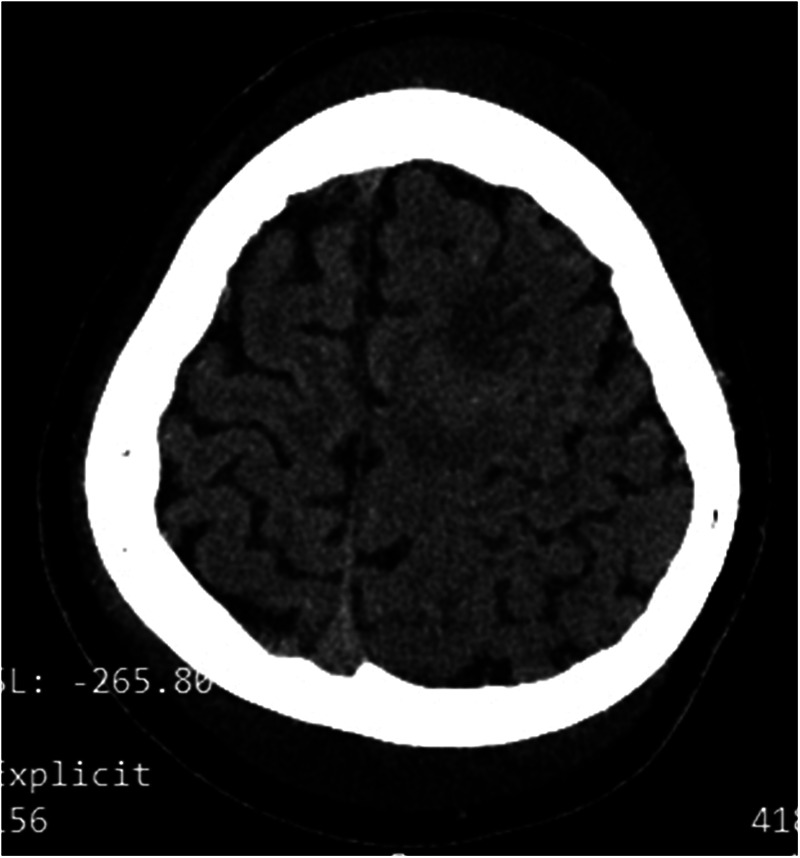
Ct revealed low density in the left frontal lobe, scalp swelling and the shallowed cerebral sulci on the left side.

**Figure 2:**
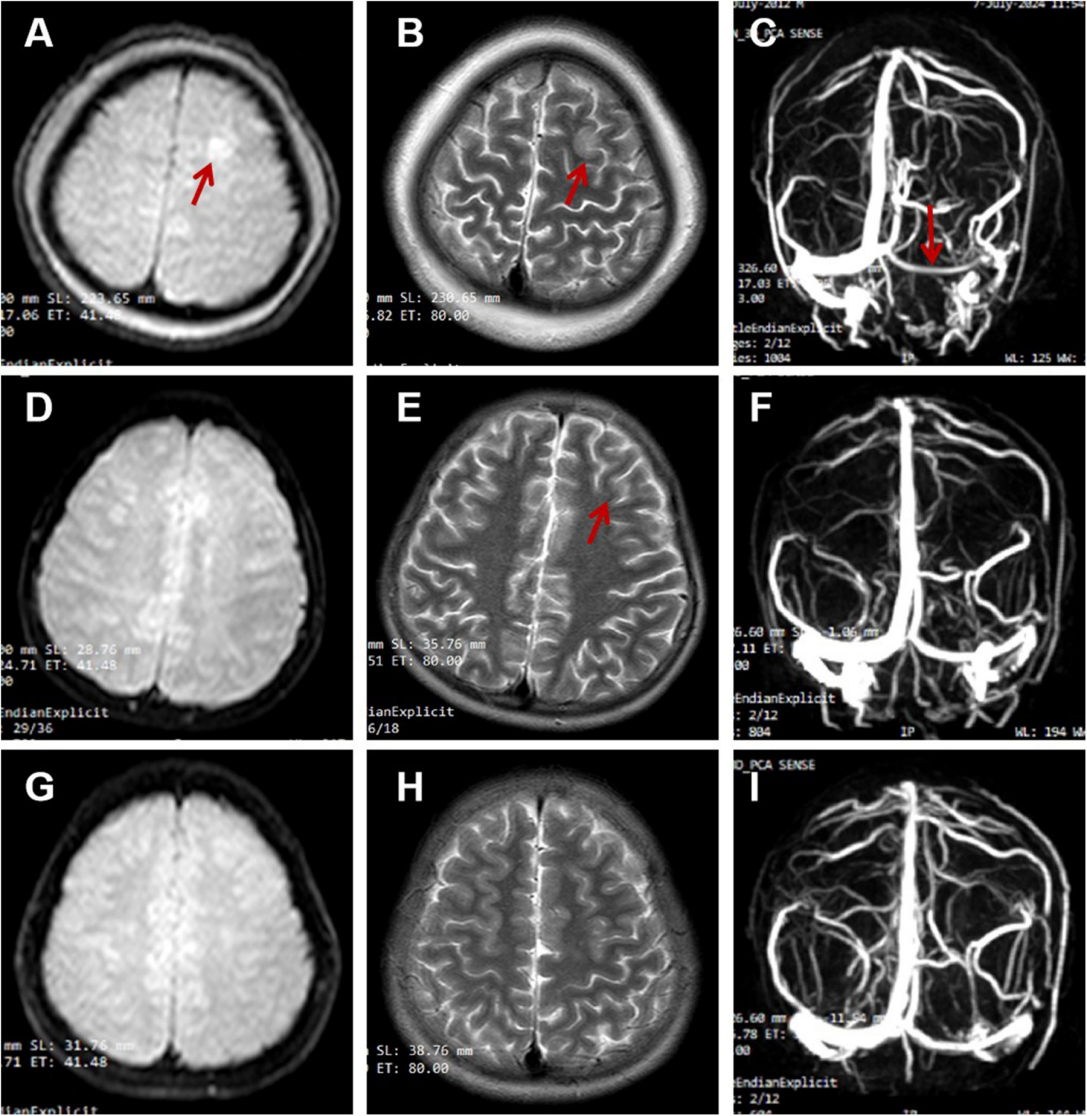
**(A–C)** on admission; **(D–F)** the second reexamination 16 days later; **(G–I)** the third reexamination 5 months later. **(A)** (DWI), **(B)** (T2-FLAIR) showed hyperintensity in the cortex and subcortex of the left frontal lobe; **(C)** (MRV) showed the reduced blood flow signal of the left transverse sinus. **(D,E)** showed the significantly shrunk of the infarct focus; **(G,H)** showed no infarct focus; **(F,I)** showed recanalization of transverse sinus.

The renal function results obtained at the local hospital 9 h before being transferred to our hospital showed a urea nitrogen level at 11.3 mmol/L, while a creatinine level at 44 μmol/L. The complete blood count showed an elevated white blood cell count at 18.75 × 10⁹/L, which consisted of 81.5% neutrophils and 14.4% lymphocytes. The red blood cell count was 4.55 × 10^12^/L, and the hemoglobin level was 13.7 g/dl with a hematocrit of 39.4%. The platelet count was 248 × 10⁹/L. An emergency head computed tomography (CT) scan in our emergency department revealed a low- density lesion in the left frontal lobe and scalp swelling. Additionally, the left cerebral sulcus was shallower than the contralateral side (Figure 1). After admission, an urgent renal function was carried out, with a urea nitrogen level of 8.36 mol/L, a creatinine level of 37.8 μmol/L. Blood chemistry analysis revealed hyperlipidemia with the level of cholesterol at 15.59 mmol/L, triglycerides at 4.61 mmol/L, high-density lipoprotein cholesterol at 3.99 mmol/L, and low-density lipoprotein cholesterol at 9.44 mmol/L, a diminished level of serum albumin at 18 g/L and IgG at 1.54 g/L. Serum sodium level was 126.1 mmol/L. It was identified that the antithrombin III level had decreased (64%, normal range 83%–128%), accompanied by an elevation of both fibrin degradation products (3.6 mg/L, normal level < 2.01) and D-dimer (1.64 mg/L, normal range 0–0.5 mg/L). The chest x-ray demonstrated the presence of bilateral lower lobe pneumonia and pleural effusion. RT-PCR for rhinovirus was positive. The assays for white blood cells, red blood cells, as well as bacteria in feces were negative results. To further differentiate central nervous system infections and autoimmune encephalitis, cerebrospinal fluid analysis, acid-fast staining, India ink staining and bacterial culture of cerebrospinal fluid, and antibodies including AMPA1, AMPA2, NMDAR, LGI1, GABABR and CASPR2 were all demonstrated to be negative. However, lumbar puncture showed that the patient's cranial pressure had increased. Cranial MRI and MRV were subsequently performed and the findings indicated abnormal signals in the cortex and subcortical area of the left frontal lobe, suggesting venous infarction (Figures 2A,B). Meanwhile, the blood flow signal of the left transverse sinus was decreased which suggesting sinus venous thrombosis (Figure 2C). To evaluate potential hypercoagulable states, anticardiolipin antibodies, lupus anticoagulant, and anti-β2 glycoprotein antibodies were assessed, all yielding negative results. Due to limited resources, the following thrombophilia-related factors were unavailable: protein S and C activity assays, methylenetetrahydrofolate reductase (MTHFR) gene mutation analysis, factor V Leiden (FVL) testing, prothrombin G20210A mutation screening, and plasminogen activator inhibitor-1 (PAI-1) activity measurement. Due to the emergent nature of the admission, initial urine sampling was deferred. Subsequent urinalysis revealed 3 + proteinuria, with quantitative measurement on day 2 demonstrating protein excretion of 2.77 g (70 mg/kg/day). The video electroencephalogram (EEG) examination demonstrated no abnormal findings.

The patient was immediately initiated on high-flow nasal cannula oxygen therapy and subcutaneous LMWH at 100 U/kg/day. Based on MRI and MRV findings, the LMWH dosage was adjusted to 100 U/kg q12h. Upon admission, diagnostic evaluation confirmed hypovolemia and tissue edema. The patient received intravenous albumin and hydroxyethyl starch for volume resuscitation, followed by mannitol and furosemide for intracranial hypertension and edema management. Concurrently, the patient received adjunctive therapies including ceftriaxone for infection management, intravenous immunoglobulin, and sodium supplementation. Prednisone was continued for NS treatment. The dose of tacrolimus was adjusted to 1.5 mg q12h owing to the relatively low trough concentration of FK-506 at 1.6 ng/ml.

Notably, the patient's consciousness normalized by day 4 of antithrombotic therapy, with no subsequent seizure activity. Clinical improvement was gradually observed, including resolution of cough, diarrhea and edema, along with normalization of serum urea nitrogen and sodium levels. Anticoagulation therapy was transitioned from LMWH to rivaroxaban at a dose of 15 mg daily on day 6. Following a 9-day hospitalization, he was discharged on rivaroxaban, tacrolimus, prednisone, calcium, and vitamin D, with additional recommendations for influenza and pneumococcal vaccinations.

At the one-week post-discharge follow-up, cranial MRI and MRV demonstrated partial thrombus resolution (Figures 2D–F). The urinary protein quantification decreased to 169 mg, maintaining sustained remission except for a transient increase to 265 mg following an upper respiratory infection, during which prednisone tapering continued. Later rechecks indicated that the level of DD, FDP, AT-III, albumin, and cholesterol levels had all returned to normal (Table 1). By 3.5 months, in the absence of thrombotic risk factors and clinical symptoms, rivaroxaban was reduced to 10 mg daily. Follow-up imaging at 5 months revealed nearly complete recanalization (Figures 2G–I), prompting further dose reduction to 5 mg daily. No bleeding complications or neurological sequelae were observed during the treatment course.

**Table 1:** The patients’ laboratory findings.

Date	PT (s)	APTT(S)	Fg （g/L）	D-dimer （mg/L）	FDP （mg/L）	AT-III （%）	ALB （g/L）	BUN （mmol/L）	Chol （mmol/L）
2024.7.6 before admission								11.3	
2024.7.7 admission	9.4	25.7	4.4	1.64	3.6	64	18	8.36	15.59
2024.7.11	12.5	34.1	4.44	0.52	1.68	81	17	5.64	16.8
2024.7.16							22		
2024.7.23							34	9	9.98
2024.7.30								5.63	
2024.8.13	11.7	33.4	2.76	0.15	1.4	125		7.18	
2024.9.26	11.2	33.8	2.45	0.1	1.1	120	49	6.42	5.34
2024.12.3	12.7	35.9	2.62	0.08	1.2	129	48	8.44	4.7

PT, prothrombin time; APTT, activated partial thromboplastin time; Fg, fibrinogen; FDP, fibrin degradation products; AT-III, antithrombin III; ALB, albumin; Chol, cholesterol.

## 3 Discussion

It is recognized that NS is a state of hypercoagulability, and children suffering from it are at risk of VTE. CVST is a rare and one of the most lethal forms of VTE in children. According to current reports, the most frequently adopted regimen is short-period of unfractionated heparin or LMWH followed by long-term (3–12 months) treatment with VKAs (5). However, this regimen requires monitoring of the international normalized ratio to keep it between 2.0 and 2.5. Frequent blood tests pose a serious burden to children and their parents. DOACs, which have no need for monitoring, have been approved for the treatment and prevention of VTE in children (12, 13). A randomized, controlled, phase 3 trial, which enrolled 500 children, revealed that patients with CVST can be treated with rivaroxaban without increasing the risk of bleeding (14). However, rivaroxaban is 87% protein binding and 66% renal elimination (13). Urinary protein losses in NS may lead to the increase in the free portion of rivaroxaban, which may increase the risk of bleeding. Currently, the research on the application of rivaroxaban in VTE in children with NS is only based on case reports (9–11), and data on the use of rivaroxaban in pediatric patients with NS complicated by CVST were scarce.

The most common symptoms of CVST are headache (5). Headache represents one of the most prevalent symptoms encountered in the pediatric emergency department and establishing an accurate diagnosis is critically important. Primary headaches, exemplified by tension-type headaches and migraines, are the most common. Primary headaches usually manifest as acute recurrent or chronic non-progressive. Headaches exhibiting progressive worsening, abrupt onset with maximal intensity, or persistent exacerbation triggered by Valsalva maneuvers should raise suspicion for secondary etiologies. Additionally, the emergence of new or qualitatively distinct headache, particularly when accompanied by focal neurological deficits or seizure activity, further supports an underlying pathology rather than a primary headache disorder (15). NS could lead to increased intracranial pressure, resulting in severe headache which can wake children from their sleep, vomiting, and in more severe cases, seizure and coma. CVST was the most likely underlying cause (16). Consequently, when a patient with NS complains of headache, especially when accompanied by infections, dehydration and serum albumin levels below 20 g/L (17), CVST should be strongly considered.

We present a case of SRNS complicated by transverse sinus thrombosis and cortical venous infarction. During the non-remission phase, the patient developed respiratory/gastrointestinal infections followed by neurological symptoms (headache, recurrent seizures, and altered consciousness). Imaging confirmed transverse sinus thrombosis with cortical venous infarction. The thrombosis-induced venous outflow obstruction led to elevated venous pressure and subsequent infarction. Initial 5 days of LMWH followed by rivaroxaban resulted in thrombus resolution, with near-complete recanalization observed after 5 months of anticoagulation therapy. This study represents the first documented application of rivaroxaban in the management of pediatric patients with NS complicated by CVST.

## 4 Conclusion

Rivaroxaban is safe and effective for CVST and cortical venous infarction in children with NS. Further studies need to be carried out to explore the safety and guidelines of rivaroxaban therapy in pediatric NS. Further research is required to investigate the safety and guidelines for the use of rivaroxaban in the treatment of NS in children.

## Patient perspective

When I learned that I got NS and needed to take oral glucocorticoids, I was really stressed. My condition didn't improve during follow-ups, and the swelling coming back scared me. Dad said I had convulsions and was unconscious for days. But the doctors and nurses took great care of me. My father was informed that I needed long-term anticoagulant therapy. He couldn't accept the measures of long-term subcutaneous injections. So, the doctor prescribed rivaroxaban, an oral anticoagulant that doesn't require repeated monitoring of indicators. After treatment, I got better. My test results returned to normal, the urine protein disappeared, and the medicine doses were reduced. I'm confident now and hope my condition won't come back.

## Data Availability

The original contributions presented in the study are included in the article/Supplementary Material, further inquiries can be directed to the corresponding author.
